# Impact of storage conditions on electromechanical, histological and histochemical properties of osteochondral allografts

**DOI:** 10.1186/s12891-015-0776-y

**Published:** 2015-10-23

**Authors:** Tomas Mickevicius, Alius Pockevicius, Audrius Kucinskas, Rimtautas Gudas, Justinas Maciulaitis, Aurelija Noreikaite, Arvydas Usas

**Affiliations:** Department of Orthopaedics and Traumatology, Hospital of Lithuanian University of Health Sciences Kaunas Clinics, Kaunas, Lithuania; Pathology Center, Department of Infectious Diseases, Veterinary Academy, Lithuanian University of Health Sciences, Kaunas, Lithuania; Large Animal Clinic, Veterinary Academy, Lithuanian University of Health Sciences, Kaunas, Lithuania; Institute of Sports, Lithuanian University of Health Sciences, Kaunas, Lithuania; Institute of Physiology and Pharmacology, Lithuanian University of Health Sciences, Kaunas, Lithuania

## Abstract

**Background:**

Osteochondral allograft transplantation has a good clinical outcome, however, there is still debate on optimization of allograft storage protocol. Storage temperature and nutrient medium composition are the most critical factors for sustained biological activity of grafts before implantation. In this study, we performed a time-dependent in vitro experiment to investigate the effect of various storage conditions on electromechanical, histological and histochemical properties of articular cartilage.

**Methods:**

Osteochondral grafts derived from goat femoral condyles were frozen at −70 °C or stored at 4 °C and 37 °C in the medium supplemented with or without insulin-like growth factor-1 (IGF-1). After 14 and 28 days the cartilage samples were quantitatively analysed for electromechanical properties, glycosaminoglycan distribution, histological structure, chondrocyte viability and apoptosis. The results were compared between the experimental groups and correlations among different evaluation methods were determined.

**Results:**

Storage at −70 °C and 37 °C significantly deteriorated cartilage electromechanical, histological and histochemical properties. Storage at 4 °C maintained the electromechanical quantitative parameter (QP) and glycosaminoglycan expression near the normal levels for 14 days. Although hypothermic storage revealed reduced chondrocyte viability and increased apoptosis, these parameters were superior compared with the storage at −70 °C and 37 °C. IGF-1 supplementation improved the electromechanical QP, chondrocyte viability and histological properties at 37 °C, but the effect lasted only 14 days. Electromechanical properties correlated with the histological grading score (*r* = 0.673, *p* < 0.001), chondrocyte viability (*r* = −0.654, *p* < 0.001) and apoptosis (*r* = 0.416, *p* < 0.02). In addition, apoptosis correlated with glycosaminoglycan distribution (*r* = −0.644, *p* < 0.001) and the histological grading score (*r* = 0.493, *p* = 0.006).

**Conclusions:**

Our results indicate that quality of allografts is better preserved at currently established 4 °C storage temperature. Storage at −70 °C or at 37 °C is unable to maintain cartilage function and metabolic activity. IGF-1 supplementation at 37 °C can enhance chondrocyte viability and improve electromechanical and histological properties of the cartilage, but the impact persists only 14 days. The correlations between cartilage electromechanical quantitative parameter (QP) and metabolic activity were detected. Our findings indicate that non-destructive assessment of cartilage by Arthro-BST is a simple and reliable method to evaluate allograft quality, and could be routinely used before implantation.

## Background

Articular cartilage injury is a frequent incidental finding during knee arthroscopic surgery [1, 2]. Large osteochondral defects in the knee joint present a very big challenge to the orthopaedic surgeons and remain an important risk factor for osteoarthritis development. Among numerous treatment options currently available in clinical practice, osteochondral transplantation is the only biological technique that can anatomically and functionally restore the hyaline cartilage [3–9]. Autologous osteochondral transplantation has demonstrated good to excellent clinical results [10–13], however, its use is limited due to the lack of healthy cartilage tissue and related donor site morbidity [14, 15].

Cartilage is avascular, aneural and relatively immunoprivileged tissue populated with chondrocytes residing within the extracellular matrix, thus, making it attractive for allogeneic transplantation [16, 17]. Efficacy of osteochondral allograft transplantation (OCA) has already been previously established [18–25]. However, its application is limited by the need for infectious disease screening, which requires extended allograft storage [26, 27]. Long-term in vitro maintenance of osteochondral allograft tissue poses a very big challenge, especially for cell viability. Chondrocyte viability is a major determinant of graft performance in vivo. Implantation of viable chondrocytes can assure prolonged maintenance of the extracellular matrix and integrity of the articular cartilage after transplantation. Despite numerous studies on allograft preparation, storage conditions and already approved protocols used by tissue banks, research on how to maintain viable cells is still ongoing [18, 28]. Several studies have shown that deep freezing and cryopreservation leads to plummeted chondrocyte viability [29–31]. Hypothermic storage at 4 °C, currently used by most tissue banks, has been shown capable of maintaining chondrocyte viability and matrix integrity [32–34]. Recently allograft storage at 37 °C has been proposed, because superior results in comparison with refrigeration have been reported [29, 35].

Different storage media compositions have been used to improve chondrocyte survival during allograft storage. Serum-free medium [19, 35, 36], fetal bovine serum (FBS) supplemented medium [29, 37], dexamethasone [38], etanercept [39] and platelet-rich plasma [40–42] have been used with varying success. Insulin-like growth factor-1 (IGF-1) is also considered to be a viable treatment option for osteochondral injuries. IGF-1 stimulates chondrocyte proliferation and synthesis of the extracellular matrix, and is chondroreparative [42, 43]. These studies suggest a potential role of different biological regulators in cartilage repair, however, their appropriate dosing and impact on chondrocyte metabolic activity is unknown.

Research has shown that apoptosis or programmed cell death is crucial for maintaining the appropriate number of cells and tissue organization. Chondrocyte death by apoptosis has been linked with cartilage matrix degradation and initiation of osteoarthritic lesions [44, 45]. A substantial relationship between inhibition of chondrocyte apoptosis and improved chondrocyte viability during cold storage of osteochondral grafts has been demonstrated [46]. This study suggested that the apoptotic process could be manipulated to enhance chondrocyte survival and improve the results of osteochondral allograft transplantation. Robertson et al. have studied apoptotic gene expression in stored osteochondral allografts and found many apoptotic genes with prolonged storage [47].

Recently, a new technology has been employed to assess functional properties of the cartilage. Arthro-BST, an arthroscopic device, enables measurement of cartilage streaming potentials, which have been emphasized as an important parameter of cell and matrix viability [48]. Streaming potentials reflect cartilage composition and function, and are sensitive to degradative changes. It has been shown that the electromechanical measurements obtained with arthro-BST correlate with histological scores and biomechanical parameters, and provide rapid and reliable assessment of articular cartilage damage [49].

Progress in tissue transplantation, increased availability of fresh donor tissue, and greater demand, especially in athletic and aging population, may explain the growing need for allograft tarnsplantation procedures, which speed up recovery and help return to sports, or postpone arthroplasty for the elderly. However, the lack of clinical evidence on best preservation conditions for OCA requires more investigation on retaining chondrocyte viability and cartilage matrix integrity during allograft screening. Different storage conditions have to be re-evaluated and an optimal allograft tissue storage protocol must then be proposed.

The purpose of this study was to assess electromechanical, histological and histochemical properties of articular cartilage after allograft storage for 14 and 28 days at −70 °C, 4 °C and 37 °C, and investigate the effect of IGF-1 supplementation at 4 °C and 37 °C. Secondarily, we aimed to examine the relationship between cartilage streaming potentials, measured by Arthro-BST device, and chondrocyte viability, apoptosis and histological evaluation.

## Methods

### Osteochondral graft preparation and storage

**Fig. 1:**
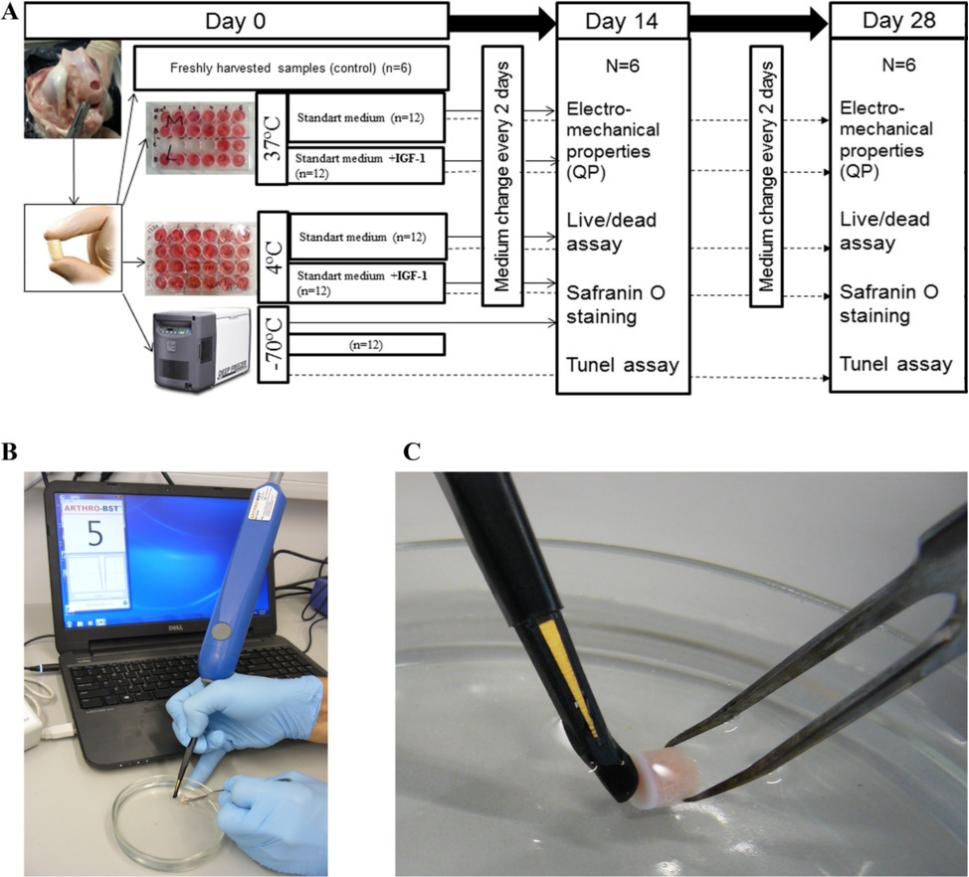
Sketch of the experimental setup. **a** The design of experimental setting showing different allograft storage conditions and evaluation time points. **b**, **c** In vitro evaluation of the graft electromechanical properties with Arthro-BST

All experiments were approved by the Animal Health and Welfare Department, State Food and Veterinary Service. Osteochondral plugs, consisting of articular cartilage and subchondral bone (5 mm diameter), were harvested from medial femoral condyles of six 5 to 6-month old male Saanen goats using standard OATS technique (Arthrex Inc, USA) under sterile conditions. The samples were rinsed with phosphate-buffered saline (PBS; Sigma) supplemented with 100 U/mL penicillin, 100 μg/mL streptomycin and 0.25 μg/mL fungizone (PSF) (Sigma), and randomly allocated to the following groups (*n* = 6 in each group): freshly harvested control (Fresh), frozen at −70 °C (−70 °C), refrigerated at 4 °C in 100 % atmospheric air (4 °C), and incubated at 37 °C (95 % humidity, 5 % CO_2_) (37 °C). The samples from 4 °C and 37 °C groups were placed in 24-well plates containing low glucose Dulbecco's Modified Eagle's Medium (DMEM) (Sigma), supplemented with 10 % fetal bovine serum (FBS) (Sigma), 1 % of PSF), 2 mM L-glutamine (Sigma), 25 μg/mL L-ascorbic acid (Sigma), and 0.1 mM MEM non-essential amino acids solution (Sigma) with or without 100 ng/mL of IGF-1(Sigma). The samples were stored for 14 or 28 days. At the end of storage period, the osteochondral plugs were first analyzed for electromechanical properties, and then cut in half and subjected to further processing for evaluation of chondrocyte viability, apoptosis, glycosaminoglycan expression and histological scoring. The freshly harvested control samples were analyzed on the same day after collection (Fig. 1).

### Electromechanical measurements

The electromechanical properties of the samples stored at different temperatures and nutritive medium conditions were evaluated with Arthro-BST™ (Biomomentum Inc., Laval, QC, Canada). The Arthro-BST is a relatively new medical device for non-destructive evaluation of articular cartilage electromechanical properties. The hand-held hemispherical indenter containing an array of 37 gold microelectrodes (5 microelectrodes/mm^2^) records streaming potentials in the articular cartilage generated during gentle compression. During compression positive mobile ions in the interstitial fluid are displaced relative to the fixed negatively charged proteoglycan molecules, which are entrapped in the collagen network creating normal streaming potentials. In degenerated cartilage the network of collagen and proteoglycans is degraded leading to very low streaming potentials. A quantitative parameter (QP, arbitrary units), calculated by the computer software, corresponds to the number of microelectrodes in contact with the cartilage when the sum of streaming potentials reaches 100 mV. High QP indicates high compliance and, therefore, weak electromechanical properties reflecting poor load-bearing capacity of the cartilage. On the contrary, low QP indicates strong electromechanical properties and high load-bearing capacity [49]. Prior to testing, the ostochondral grafts were immersed in PBS solution for 20 min and the QP of each graft was recorded 5 times in order to obtain median values.

### Detection of chondrocyte viability

Chondrocyte viability was analyzed using LIVE/DEAD® assay (Molecular Probes, Eugene, OR, USA) staining and confocal microscopy. The samples were washed in PBS and then labelled with 1 μM calcein AM (to detect live cells) and 2 μM ethidium homodimer-1 (to detect dead cells). Thereafter, the samples were incubated for 40 min at 37 °C, washed 3 times in PBS for 10 min and processed for imaging. 200 μm thick slices were imaged along the vertical profile with inverted confocal microscope LSM 700 Axio Observer Z.1 (Carl Zeiss, Germany). Image stacks, consisting of 8 slices each at 0.74 μm interval were obtained at 20x magnification. Live and dead cells were counted in 2 random areas (200 × 400 μm) in each tissue section using Image J version 1.47q (National Institutes of Health, Bethesda, MD, USA). Chondrocyte viability was calculated as a percentage of live cells relative to the total cell number.

### Histological evaluation

The samples were fixed in 10 % neutral buffered formalin, decalcified in Shandon TBD-2 Decalcifier (TBD; Thermo Scientific, Kalamazoo, MI, USA) and embedded in paraffin. Cartilage sections (5 μm thick) were stained with safranin O-fast green using an established protocol [50] to detect the glycosaminoglycan (GAG) positive matrix that is typical for the hyaline cartilage and normally stains red. Light microscopy images were taken at 4× magnification with Olympus BX63 microscope (Olympus, Japan) equipped with Olympus DP72 CCD camera using CellSens Dimension imaging software (Olympus, Japan). The images were analysed using Image J software. Previously described image processing protocol was adapted to quantify the amount of GAG distribution in each section [51]. Briefly, red colour intensity (RCI) was calculated in the entire cross-sectional slice area by obtaining red, green, and blue (RGB) image planes in a scale of 256 values (black = 0). The fraction of red (RF) was defined as the ratio of the R component to the sum of the R, G, and B components: RF = R/(R + G + B) and expressed as a percentage. Sections were additionally evaluated by 3 blinded observers using histological-histochemical grading system proposed by Mankin et al. [52]. Fresh articular cartilage received the score of 0, whereas higher score indicated more deteriorated cartilage.

### TUNEL assay for chondrocyte apoptosis

Apoptosis was evaluated with TUNEL assay kit (ApopTag® Peroxidase In Situ Apoptosis Detection Kit; Millipore, USA) using the protocol provided by the manufacturer. Briefly, deparaffinised tissue sections were incubated in 20 μg/mL Proteinase K (Ambion) for 15 min at room temperature. After endogenous peroxidase has been quenched by incubation in 3 % hydrogen peroxide for 5 min, the ends of fragmented DNA in the tissue were labelled with terminal deoxynucleotidyl transferase (TdT) enzyme in the presence of digoxigenin-conjugated nucleotides and unlabeled nucleotides for 60 min in humidified chamber at 37 °C. The slides were then incubated for 30 min with anti-digoxigenin antibodies conjugated to a peroxidase reporter molecule. Immunohistochemical detection of these antibodies was carried out by exposure to a chromogenic DAB substrate (Sigma) for 10 min. The slides were counterstained with Methyl Green (Sigma) solution for 5 min, rinsed in distilled water, dehydrated in alcohol, cleared in xylenes, and mounted with cytoseal under a coverglass.

High magnification images (200×) were obtained with Olympus BX63 microscope (Olympus, Japan). The number of apoptotic (brown-black) and viable (green) cells was counted in 3 random areas (200 × 400 μm) selected in two tissue sections from each sample. The percentage of apoptotic cells was then calculated.

#### Statistical analyses

The data were analyzed using the SPSS v.19. All results are presented as the mean ± standard deviation. Statistical differences between the sample groups were assessed using one-way repeated measures analysis of variance (ANOVA) with Bonferroni post hoc multiple comparison test. *p* < 0.05 was considered statistically significant. The relationship between the QP, chondrocyte viability, apoptosis, GAG distribution and histological grading scores was assessed by nonparametric correlation analysis using Spearman‘s correlation coefficient. A significant correlation was present when *p* < 0.05.

## Results

### Electromechanical properties of osteochondral grafts

**Fig. 2:**
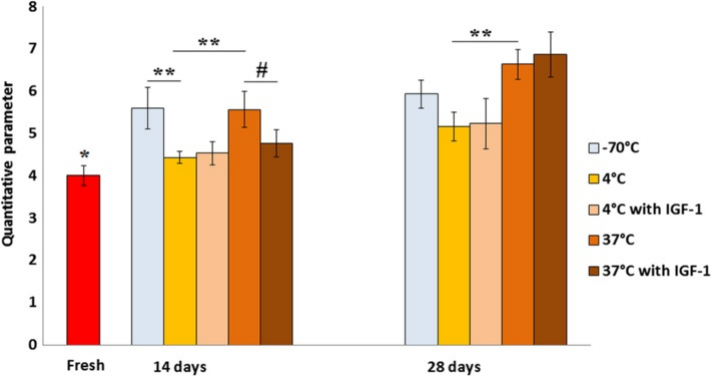
Electromechanical properties of osteochondral samples, as analyzed by Arthro-BST. Electromechanical properties of samples stored in different temperature and nutritive medium conditions were evaluated after 14 and 28 days. Freshly harvested cartilage was used as control. Quantitative parameter (QP) measurements are presented as arbitrary units (mean ± standard deviation). *p < 0.001 (fresh versus −70 °C and 37 °C group after 14 days, and versus all other groups after 28 days); **p < 0.001; ^#^ *p* < 0.05

The QP of the freshly harvested samples on average was 4 ± 0.23 (Fig. 2). We observed significantly increased QP after 14 days for the samples stored at −70 °C or incubated in DMEM at 37 °C (5.6 ± 0.49 and 5.57 ± 0.43, respectively; *p* < 0.001). For the samples stored in DMEM at 4 °C, we detected a slightly increased QP (4.43 ± 0.15), however, QP was significantly lower compared to both aforementioned groups (*p* < 0.001). When the culture medium was supplemented with IGF-1, there was a slight increase of QP for the grafts stored at 4 °C (4.53 ± 0.27), however, a considerable decrease of QP was detected for the grafts stored at 37 °C (4.77 ± 0.32; *p* = 0.043).

After 28 days of storage, electromechanical properties of the samples worsened. For the samples stored at −70 °C, only a slight increase of QP (5.93 ± 0.33) was detected. For the grafts incubated in DMEM at 37 °C, we observed a significant increase of QP (6.63 ± 0.34; *p* = 0.001) when compared to 14-day storage period. Although there was a diminutive increase of QP in the refrigeration group (5.17 ± 0.34), it was significantly lower compared to the incubation at 37 °C group (*p* < 0.001). The addition of IGF-1 to the culture medium at this time had no significant effect on QP at 4 °C and 37 °C. These findings clearly indicate that allograft refrigeration has a greater capacity of preserving electromechanical properties of the osteochondral grafts compared to freezing or incubation at 37 °C and could prevent rapid deterioration of the cartilage.

### Chondrocyte viability

**Fig. 3:**
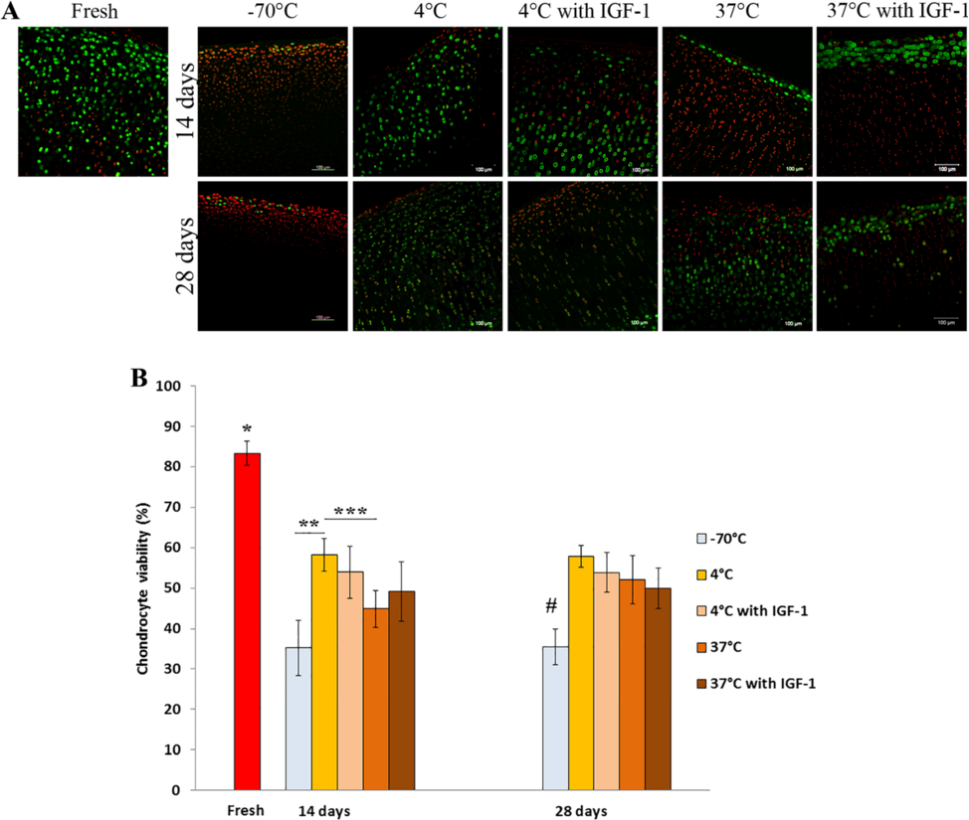
Chondrocyte viability in osteochondral samples, as analyzed by LIVE/DEAD® fluorescence staining. **a** Confocal microscopy images showing abundance of live cells (*green*) in fresh cartilage and increased population of dead cells (*red*) after 14 and 28 days of storage in different conditions. Scale bar = 100 μm. **b** Chondrocyte viability was calculated as a percentage of live cells to the total number of cells (mean ± standard deviation). *p ≤ 0.001 (fresh versus all other groups after 14 and 28 days); **p < 0.001; ***p = 0.003; ^#^ *p* ≤ 0.001(−70 °C group versus all other groups after 28 days)

Chondrocyte viability for the freshly harvested articular cartilage was approximately 83.3 ± 2.98 % (Fig. 3a, b). We detected a significant decrease of chondrocyte viability in all the storage groups after 14 and 28 days (*p* < 0.001) compared to fresh cartilage samples. The samples stored at −70 °C contained smallest amount of viable chondrocytes compared with any other storage group. After 14 days, the −70 °C samples contained only 35.2 ± 6.9 % of viable chondrocytes, whereas samples stored in DMEM medium at 37 °C contained 44.87 ± 4.53 % of viable chondrocytes. No significant difference was detected between these two groups. For the samples stored in DMEM at 4 °C, viability was 58.22 ± 4.07 % and it was significantly higher compared with the −70 °C and 37 °C storage groups (*p* < 0.001 and *p* < 0.003, respectively). When IGF-1 was added to the medium, chondrocyte viability decreased in the 4 °C storage group (53.89 ± 6.48 %) and increased in the 37 °C storage group (49.22 ± 7.35 %), but this change was not significant.

After 28 days, the samples stored at −70 °C contained significantly less viable chondrocytes (35.46 ± 4.36 %) compared with any other storage group (*p* ≤ 0.001). There was no significant difference in chondrocyte viability between the samples stored at 37 °C and 4 °C (52.02 ± 5.97 % and 57.80 ± 2.71 %, respectively). When IGF-1-supplemented medium was used, chondrocyte viability slightly decreased in the 4 °C and 37 °C groups (53.81 ± 4.90 % and 49.93 ± 4.96 %, respectively). These results suggest that storage at −70 °C is unable to maintain chondrocyte viability. Storage at 4 °C is capable of maintaining significantly higher chondrocyte viability than storage at 37 °C, but this effect lasts only 2 weeks. IGF-1 supplementation increased chondrocyte viability at 37 °C during 14-day storage, but did not reveal a profound effect at any time at 4 °C and 37 °C after 28 days.

### GAG quantification by safranin O staining

**Fig. 4:**
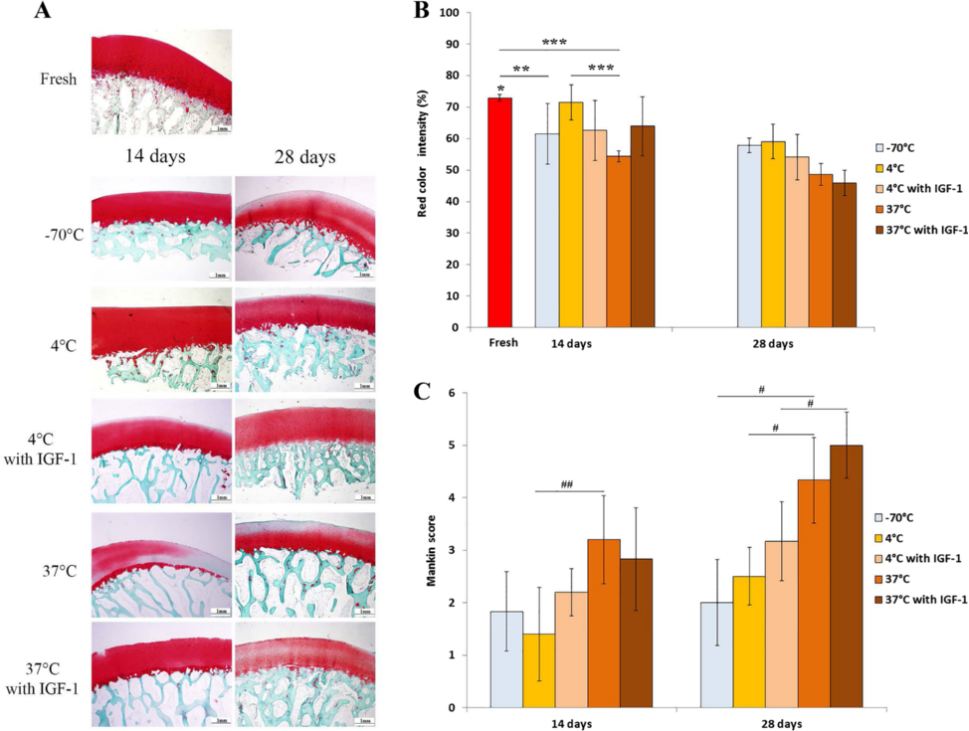
GAG distribution in osteochondral samples, as analyzed by Safranin O-Fast Green staining, and histological–histochemical grading according to Mankin. **a** Histological images of fresh cartilage and after 14 and 28 days of storage in different conditions. Scale bar = 1 mm. **b** Quantitative analysis of GAG distribution by red color intensity (RCI) using an adapted image processing protocol. All samples revealed significantly reduced RCI after 28 days of storage. *p < 0.002 (fresh versus all other groups after 28 days); **p < 0.02; ***p ≤ 0.001 (**c**) Histological-histochemical grading scores. Fresh cartilage was assigned with the score of 0, while higher score indicates cartilage deterioration. ^##^ *p* < 0.02; ^#^ *p* = 0.005

Cartilage sections from all sample groups were stained with safranin O-fast green and evaluated for GAG distribution (Fig. 4a). Red colour intensity (RCI) of the freshly extracted normal articular cartilage was 72.95 ± 1.06 % (Fig. 4b). We observed a significant reduction of RCI after storage at −70 °C for 14 days (61.50 ± 9.60 %; *p* = 0.018). RCI of samples stored in DMEM at 4 °C remained very close to a fresh cartilage level 71.48 ± 5.61 %. When IGF1 was added to the medium at 4 °C RCI decreased to 62.58 ± 9.57 %, but this change was not significant. For the samples stored in DMEM at 37 °C, we observed a significant reduction of RCI compared with the fresh and refrigerated samples (54.4 ± 1.76 %; *p* ≤ 0.001). When IGF-1 was added to the medium at 37 °C, RCI increased to 63.98 ± 9.35 %, but this increase was not significant. After 28 days, RCI for all the samples was significantly lower compared with the fresh samples and constituted 57.89 ± 2.31 %, 59.02 ± 5.46 % and 48.58 ± 3.43 %, for −70 °C, 4 °C and 37 °C storage groups, respectively (*p* < 0.002). A significant reduction of RCI compared with a 14-day storage period was detected in the samples stored in DMEM at 4 °C (*p* = 0.078). When IGF-1 was added to the storage medium at 4 °C and 37 °C, RCI decreased to 54.12 ± 7.27 % and 45.91 ± 4.1 %, respectively. There was no statistically significant difference between the groups after 28 days. These results indicate that allograft storage at 4 °C maintains GAG distribution near the normal levels for 14 days and is superior compared with storage at 37 °C. IGF-1 supplementation did not help to maintain GAG distribution at 4 °C, but facilitated these properties at 37 °C. However, during extended storage GAG distribution decreased and there was no significant difference between the groups.

### Histological grading

We also analyzed whether different storage conditions had effect on histopathology. We used the histological-histochemical grading system proposed by Mankin et al. [52]. Even though this method is intended to be used for the grading of the human osteoarthritic cartilage, it has also been used in animal studies [53, 54]. After 14 days, the samples stored at −70 °C and 4 °C exhibited slightly reduced safranin O staining with diffuse hypercellularity at the superficial cartilage layer and received the scores of 1.83 ± 0.75 and 1.4 ± 0.89, respectively (Fig. 4c). In contrast, the samples stored at 37 °C received a score of 3.2 ± 0.84 because of structural irregularities, hypocellularity and clonal cells, which were accompanied by a slight reduction in safranin O staining throughout the superficial and middle layer of the cartilage. In addition, the histological score in the 37 °C group was significantly higher (*p* = 0.019) compared with the 4 °C group, indicating more rapid cartilage deterioration. The histological scores after 28 days increased in each group. The samples stored at 37 °C received significantly higher scores compared with the −70 °C and 4 °C groups (4.33 ± 0.82 versus 2.0 ± 0.82 and 2.5 ± 0.55, respectively; *p* = 0.005), as was evident by a more diffuse hypercellularity and markedly reduced safranin O staining throughout the samples.

IGF-1 addition to the medium at 4 °C and 37 °C had no profound effect on the histological scores compared with DMEM alone after 14 days of storage, although a slight improvement was detected at 37 °C. Higher histological scores were observed after 28 days, but significant increase compared with the 14-day period was detected only in the 37 °C group (*p* < 0.001) after IGF-1 supplementation. Increased diffuse hypercellularity was evident in both IGF-1 supplemented groups after prolonged storage. In addition, inferior surface structure, moderate to severe reduction in safranin O staining and clonal cellularity with hypocellular areas were notable throughout the 37 °C samples after 28 days of storage.

### Chondrocyte apoptosis

**Fig. 5:**
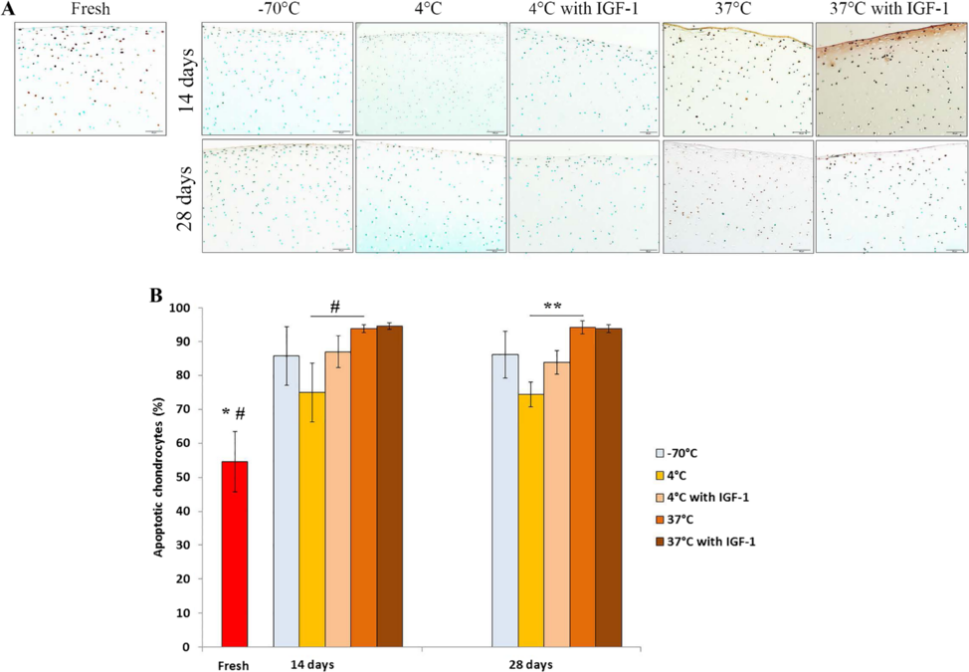
Chondrocyte apoptosis in osteochondral samples, as analyzed by TUNEL immunoassay to detec apoptotic cells. **a** Light microscopy images showing abundance of viable cells (green) in fresh cartilage and increased population of apoptotic cells (brown-black) after 14 and 28 days of storage in different conditions. Scale bar = 50 μm. **b** The percentage of apoptotic cells was calculated as the number of apoptotic cells related to the total number of cells. Apoptosis significantly increased throughout different storage groups. *p < 0.001 (fresh versus all other groups, except 4 °C after 14 and 28 days); #*p* < 0.05 (fresh versus 4 °C after 14 and 28 days); **p = 0.008

The extent of chondrocyte apoptosis varied among different storage groups. The freshly harvested samples contained on average 54.55 ± 8.87 % of TUNEL positive chondrocytes (Fig. 5). After 14 days, a significant increase in apoptotic chondrocyte number was observed at −70 °C and 37 °C (85.82 ± 8.72 % and 93.91 ± 1.1 %, respectively; *p* < 0.001). Albeit it was a significant increase in the 4 °C group (74.98 ± 8.67 %; *p* = 0.021), this group contained considerably less (*p* = 0.015) apoptotic chondrocytes compared with the 37 °C storage group. IGF-1 supplementation had no profound effect on apoptosis at 4 °C and 37 °C.

After 28 days, the percentage of apoptotic chondrocytes in each group did not change significantly when compared with the 14-day storage. Yet again, we detected significantly less apoptotic chondrocytes in the refrigeration group compared with the 37 °C storage group (74.39 ± 3.60 % and 94.32 ± 1.90 %, respectively; *p* = 0.008). IGF-1 addition at this time also did not altered apoptosis at 4 °C and 37 °C. These findings indicate that allograft storage at 4 °C is associated with a lesser extent of apoptosis than storage at 37 °C. IGF-1 had no impact on chondrocyte apoptosis in any group at any time.

### Correlations between different quantitative parameters

**Fig. 6:**
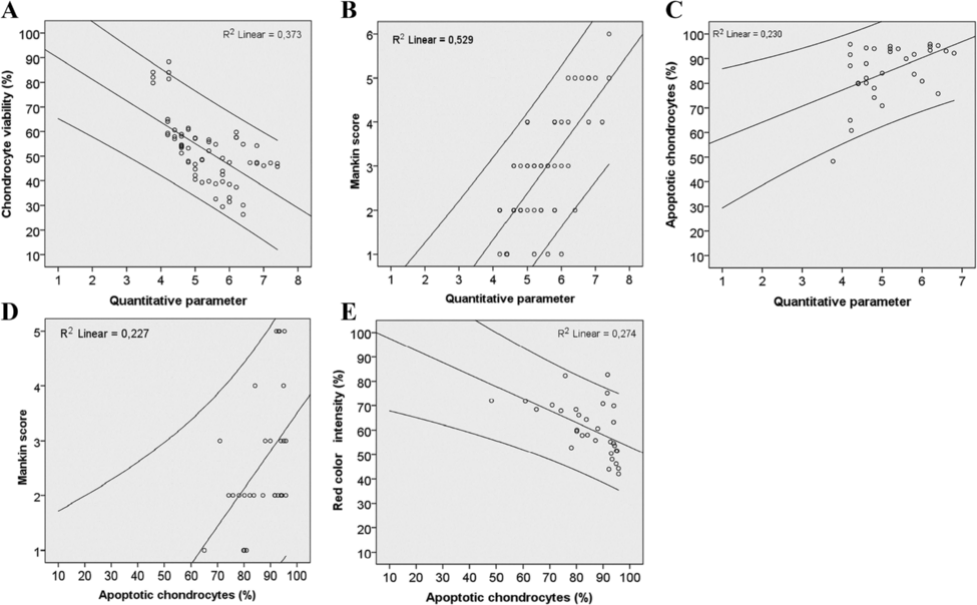
Correlations between different quantitative parameters. Bivariate linear correlations were assessed using Spearman‘s correlation coefficient. **a** Inverse correlation between chondrocyte viability (%) and quantitative parameter (QP). Correlation coefficient (*r* = −0.654), corresponding P-value (*p* < 0.01); (**b**) Direct correlation between Mankin score and QP. Correlation coefficient (*r* = 0.673), corresponding P-value (*p* < 0.01); (**c**) Direct correlation between apoptotic chondrocytes (%) and QP. Correlation coefficient (*r* = 0.416), corresponding P-value (*p* < 0.01); (**d**) Direct correlation between Mankin score and apoptotic chondrocytes (%). Correlation coefficient (*r* = 0.493), corresponding P-value (*p* < 0.01); (**e**) Inverse correlation between red color intensity (%) and apoptotic chondrocytes (%). Correlation coefficient (*r* = −0.644), corresponding P-value (*p* < 0.01)

The statistical analysis revealed that the electromechanical QP correlated directly with the histological grading score (*r* = 0.673, *p* < 0.001), apoptotic chondrocytes (*r* = 0.416, *p* = 0.018), and inversely with chondrocyte viability (*r* = −0.654, *p* < 0.001) (Fig. 6). In addition, the extent of apoptosis correlated directly with the histological grading score (*r* = 0.493, *p* = 0.006) and inversely with GAG distribution manifested by RCI measurement (*r* = −0.644, *p* < 0.001)

## Discussion

Our results demonstrate that the cartilage samples stored at 4 °C possessed higher chondrocyte viability and GAG distribution, lower apoptosis and nearly normal electromechanical properties compared with the samples stored at 37 °C. These improvements were apparent throughout 28 days. However, there was no significant difference in chondrocyte viability and GAG expression between two groups after 28 days. Our results did not support previous studies which indicate that allograft storage at 37 °C is superior to storage at 4 °C. Garrity et al. found that osteochondral allograft viability, matrix content, composition and biomechanical properties were maintained at ‘fresh’ levels through 56 days of storage in the standard medium at 37 °C, but allografts stored at 4 °C were unable to maintain viability or matrix integrity through 28 days of storage [35]. In distinction to the latter study, which used a serum-free medium, we used 10 % FBS-supplemented medium. Numerous studies have demonstrated that addition of FBS to the nutrient media can significantly increase chondrocyte survival during storage at 4 °C [32, 37, 43]. Pallante et al. reported that chondrocyte viability was higher after storage at 37 °C, instead of 4 °C, while cartilage thickness, GAG and collagen content were maintained at both temperatures [29]. In that study 4 °C samples were stored in 10 % FBS-supplemented medium pre-equilibrated to 5 % CO_2_ and tightly sealed. By contrast, in this study the specimens were stored in 24-well plates exposed to 100 % atmospheric air with medium change three times per week. Prior studies have also suggested that replenishment of nutrients by regular changes of the medium may enhance chondrocyte survival in hypothermically stored cartilage specimens [32, 55].

We also detected that storage at −70 °C was associated with significant decline in chondrocyte viability and worse electromechanical properties compared to storage at 4 °C. These results support previous findings that deep freezing leads to dramatic loss of viable chondrocytes [29]. In fact, our results conflict previous findings that storage at −80 °C does not change the mechanical properties of articular cartilage [19, 55–57]. Even though we did not assess biomechanical properties of the grafts, recent studies demonstrated significant correlation between electromechanical and biomechanical parameters [49, 58].

There is a limited number of studies that have investigated the IGF-1 effect on chondrocyte viability during allograft storage. Teng et al. [43] used a serum-free medium at 4 °C and reported that viability after 3 weeks was significantly higher in DMEM + IGF-1 group compared with DMEM alone group. However, after 4 weeks researchers observed near a 10-fold decrease in the DMEM + IGF-1 group compared with a 2-fold decrease in the DMEM alone group. Several reports have indicated that IGF-1 can decrease chondrocyte apoptosis induced by traumatic compression or collagenases treatment [59, 60]. In this study the addition of IGF-1 to DMEM at 4 °C did not produce a favourable effect on chondrocyte viability, apoptosis and GAG distribution, but the electromechanical properties in both groups were quite similar and remained near normal levels after 14 and 28 days. Interestingly, it was found that IGF-1 supplementation at 37 °C had a positive effect on chondrocyte viability, GAG distribution and significantly improved the electromechanical QP. However, these improvements lasted only for 14 days and eventually, after 28 days, all the parameters became worse compared with the samples stored in DMEM without IGF-1.

Non-destructive evaluation of electromechanical properties by Arthro-BST is a relatively new technique in cartilage research and it has already been shown very useful for quantitative assessment of functional properties in cartilage repair studies [48, 49, 58]. It has been reported that electromechanical measurements reflect cartilage material properties and are even more sensitive to the changes than biomechanical testing [58]. Although we did not detect correlation between electromechanical QP and GAG distribution (*p* = 0.059), our findings are consistent with other studies demonstrating that the electromechanical QP correlated directly with the Mankin score [49, 61]. Cartilage cellular impairment and subsequent reduction of safranin-O staining was reflected by increased QP values. In addition, it was observed that the electromechanical properties correlated with the chondrocyte viability and apoptosis as reflected by relatioship between higher QP, reduced viability and increased apoptosis. To our knowledge, this is the first report when electromechanical properties of the articular cartilage were linked with these biological parameters.

It has been reported that chondrocyte death by apoptosis is closely associated with cartilage matrix degradation which is crucial for osteoarthritis development [44]. Decreased cell viability and increased apoptosis was also detected after a traumatic joint injury [62]. Although we did not observe correletion between viability and apoptosis (*p* = 0.051), we found that apoptosis correlated directly with Mankin score and inversely with proteoglycan distribution as assessed by safranin-O staining. These findings are consistent with previous reports indicating significant linear relationship between the histopathological grade of cartilage and chondrocyte apoptosis [44, 63, 64].

One limitation of this study could be relatively small number of samples per experimental group. However, only one subjective parameter of histological grading and four quantitative evaluation parameters used in our study confirm reliability of the obtained data. In fact, the correlation analyses performed at each designated timepoint revealed similar relationship between aforementioned parameters after 14 and 28 days. To further investigate correlations between different storage groups more samples would be required. Another limitation related to our study was previously descibed by Sim et al. [49]. Although this study found no correlation between electromechanical QP and human cartilage thickness, authors indicated that it could be difficult to distinquish high electromechanical properties of the cartilage for low QP (<4) due to the geometry of Arthro-BST indenter and its limitations for thin cartilage. Regardless the fact that the goat knee articular cartilage is substantialy thinner compared to human [65], like in their study, this limitation caused no problem for us since the lowest average QP was 4.

## Conclusions

Our results indicate that the quality of stored allografts is better preserved at currently established refrigeration temperatures compared with the storage at very low temperatures or incubation at 37 °C. IGF-1 supplementation at 37 °C can enhance chondrocyte viability, improve electromechanical and histological properties of the cartilage, but the effect is diminished beyond 14 days. The non-destructive evaluation of cartilage by the Arthro-BST enables a rapid, convenient and reliable way to estimate allograft quality before implantation and warrants ordinary use of this device in assessing articular cartilage damage during arthroscopic surgery. In addition, this method could provide precise information regarding efficacy of surgical cartilage repair and is a promising technique for monitoring the outcome of regenerative processes.
